# Patterns of Dolphin Bycatch in a North-Western Australian Trawl Fishery

**DOI:** 10.1371/journal.pone.0093178

**Published:** 2014-04-02

**Authors:** Simon J. Allen, Julian A. Tyne, Halina T. Kobryn, Lars Bejder, Kenneth H. Pollock, Neil R. Loneragan

**Affiliations:** 1 Murdoch University Cetacean Research Unit, Centre for Fish, Fisheries and Aquatic Ecosystems Research, School of Veterinary and Life Sciences, Murdoch University, Murdoch, Western Australia, Australia; 2 Department of Biology, North Carolina State University, Raleigh, North Carolina, United States of America; Aristotle University of Thessaloniki, Greece

## Abstract

The bycatch of small cetaceans in commercial fisheries is a global wildlife management problem. We used data from skippers' logbooks and independent observers to assess common bottlenose dolphin (*Tursiops truncatus*) bycatch patterns between 2003 and 2009 in the Pilbara Trawl Fishery, Western Australia. Both datasets indicated that dolphins were caught in all fishery areas, across all depths and throughout the year. Over the entire datasets, observer reported bycatch rates (n = 52 dolphins in 4,124 trawls, or 12.6 dolphins/1,000 trawls) were ca. double those reported by skippers (n = 180 dolphins in 27,904 trawls, or 6.5 dolphins/1,000 trawls). Generalised Linear Models based on observer data, which better explained the variation in dolphin bycatch, indicated that the most significant predictors of dolphin catch were: (1) vessel - one trawl vessel caught significantly more dolphins than three others assessed; (2) time of day – the lowest dolphin bycatch rates were between 00:00 and 05:59; and (3) whether nets included bycatch reduction devices (BRDs) - the rate was reduced by ca. 45%, from 18.8 to 10.3 dolphins/1,000 trawls, after their introduction. These results indicated that differences among vessels (or skippers' trawling techniques) and dolphin behavior (a diurnal pattern) influenced the rates of dolphin capture; and that spatial or seasonal adjustments to trawling effort would be unlikely to significantly reduce dolphin bycatch. Recent skipper's logbook data show that dolphin bycatch rates have not declined since those reported in 2006, when BRDs were introduced across the fishery. Modified BRDs, with top-opening escape hatches from which dolphins might escape to the surface, may be a more effective means of further reducing dolphin bycatch. The vulnerability of this dolphin population to trawling-related mortality cannot be assessed in the absence of an ongoing observer program and without information on trawler-associated dolphin community size, broader dolphin population size and connectivity with adjacent populations.

## Introduction

Demersal trawl fishing for crustaceans, cephalopods and fish impacts benthic habitats and results in large quantities of incidental catch, or bycatch, of non-targeted species [1]–[3]. Trawling, gill netting and purse seining are the three largest causes of fisheries-related small cetacean mortalities worldwide [4]–[7]. Entanglements in fishing gear and large-scale habitat modification have resulted in the extinction of the Yangtze river dolphin (*Lipotes vexillifer*), representing the first loss of a cetacean species directly attributable to human influences [8]. Several other populations and, indeed, species of small cetaceans, such as the Maui's dolphin (*Cephalorhynchus hectori maui*) of New Zealand's North Island and the vaquita (*Phocoena sinus*) of the Sea of Cortez, are at risk of extinction from the cumulative impacts of fishing related mortality and disturbance from gill netting and trawl fisheries [9]–[12].

As a result of suspected and/or measured declines in dolphin populations due to fisheries bycatch, public concerns and pressure from non-government organizations, major changes in fisheries policy and practice have been implemented in several regions. After the introduction of the *U.S. Marine Mammal Protection Act* (*MMPA*) in 1972, for example, high observer coverage and a variety of bycatch mitigation measures were implemented to quantify and reduce the bycatch of two dolphin species (spotted, *Stenella attenuata*, and spinner dolphins, *S. longirostris*) in the purse seine fishery for tuna in the eastern tropical Pacific [13] [14]. Although massive reductions in dolphin capture rates were achieved, the impacted populations have not recovered [15] [16].

New Zealand's Department of Conservation administers their *MMPA* of 1978. Numerous protected areas have been established, with time- and area-based restrictions placed on fishing activities that present high entanglement risks to marine mammals. One such protected area was established off the west coast of the North Island to reduce entanglements of the critically endangered Maui's dolphin. However, concerns remain over the efficacy of these measures, as gill netting and trawling are still allowed in certain areas [17]. Surveys of the distribution of the endangered Hector's dolphins (*C. hectori*) off the South Island suggest that restrictions on commercial gill netting protect 60% or less of the dolphin population for three months of the year [18]. New Zealand's endemic dolphin populations are predicted to continue declining under current management, driven primarily by ongoing bycatch in gill net and trawl fisheries [12].

All marine mammals in Australian waters are protected under the *Environmental Protection and Biodiversity Conservation Act* 1999. Fishers are required by legislation to report fatal and non-fatal entanglements of marine mammals to State and Commonwealth fisheries management agencies. The greatest threats to small cetaceans in Australian waters are also associated with gill netting, purse seining operations and trawl fisheries [19]. Here, the species most often affected by fishing mortality include: bottlenose (*Tursiops* spp.), common (*Delphinus delphis*), Australian snubfin (*Orcaella heinsohni*), humpback (*Sousa chinensis*) and spinner dolphins [20] [21]. Thousands of dolphins have died in commercial fishing operations over the past three decades [21], the impacts of which are impossible to quantify without baseline data on the abundance and distribution of dolphin populations across the vast majority of Australian waters [22]. The tropical waters of north-western Australia are no exception, where numerous dolphin populations are exposed to commercial fishing, as well as large-scale habitat modification through the proliferation of the oil, gas and mining industries. No population estimates exist for any species in this region [23].

Trawl fisheries operate in many regions of Australian waters [24]. The North West Shelf region of Western Australia (WA) has been trawled since the early 1970s, with the Taiwanese pair-trawl fishery catching in excess of 100,000 tons of fish, cephalopods and other invertebrates in the mid-1970s [25]. Catches declined to less than 10,000 tons per annum by the mid-1980s, when Chinese and Korean trawlers also fished the area and an experimental management regime was introduced [26]. Shortly after this new regimen commenced, the foreign fleet diminished and a domestic fishery developed [25]. Since the early 1990s, catches in the Pilbara Trawl Fishery (PTF) have fluctuated between 2,000 and 3,500 tons per annum, dropping to <1,400 in the last five years, associated with a decline in trawl effort [27].

The bycatch of a number of protected species (dolphins, sea snakes, turtles and sawfish) in the PTF was first documented in 2002 [28]. A variety of bycatch mitigation techniques were pursued, focussed primarily on reducing dolphin bycatch [27] [29]. The efficacy of acoustic deterrents, or ‘pingers’, for reducing dolphin interactions with fisheries has continued to be evaluated in this and other regions, with inconsistent results depending on the dolphin species involved, type of fishery and the type and number of pingers deployed [30]. Pingers proved ineffective in deterring bottlenose dolphins from interacting with trawl gear in the PTF [31]. Field trials of bycatch reduction devices (BRDs) resulted in a reduction in the number of dolphins landed on deck and they were made compulsory across the fishery in March 2006 [29].

The dolphins subject to bycatch in the PTF are common bottlenose dolphins (*T. truncatus*, ‘bottlenose dolphins’ hereafter) [32], a globally widespread species, occurring in tropical and temperate latitudes [33]. Bottlenose dolphins are thought to be a widely distributed in Australian pelagic waters [21] [34], mixing with and/or being replaced by Indo-Pacific bottlenose dolphins (*T. aduncus*) in shallow, coastal areas, including those of north-western Australia [23] [32]. In the *Action Plan for Australian Cetaceans*, bottlenose dolphins are listed as ‘no category assigned because of insufficient information’ [20] and very little is known of the populations off north-western Australia or, indeed, any pelagic population around Australia. Due to this broad lack of even baseline data, assessments of the status of individual bottlenose dolphin stocks, or populations, are not yet possible.

Previous studies in the PTF by the Department of Fisheries WA have been based primarily on trialling the efficacy of pingers [31] and various BRDs [29] in reducing dolphin bycatch. While some aspects of the geographical and temporal nature of incidental dolphin captures were evaluated, these assessments were based on data collected during ca. six- and 18-month trials between 2004 and 2006 [29] [31]. Here, we used six years of data from skippers' logbooks and independent observer records collected from August 2003 until September 2009 to build upon this earlier research and investigate the spatial and temporal patterns of dolphin bycatch across the PTF. We aimed, firstly, to assess the spatial, daily and seasonal data on fishing effort and dolphin bycatch, and secondly, to evaluate the effectiveness of different net designs (those with and without BRDs) in reducing dolphin bycatch.

## Materials and Methods

### Characteristics of the Pilbara Trawl Fishery

**Figure 1:**
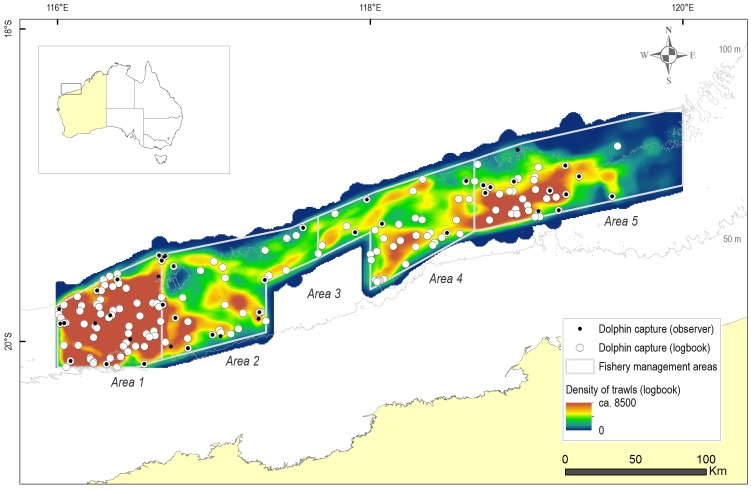
The Pilbara Trawl Fishery off the north-western coast of Australia. Figure includes: a) the 50 and 100 m depth contours; b) the boundaries of the fishery management areas (Areas 1, 2, 4 and 5; Area 3 in the central southern region of the fishery is closed to trawling); c) the spatial density of fishing effort based on logbook trawls; and d) the location of dolphin bycatch events reported in skippers' logbooks and by independent observers (August 2003 to September 2009).

The PTF, within the broader Pilbara Demersal Scalefish Fishery (which also includes trap and line fisheries), is bound by longitudes of 116° to the west and 120° to the east, and by an approximation of the 50 m depth contour to landward and the 100 m depth contour to seaward (Fig. 1). Since being gazetted in 1998, four Management Areas have been open to trawling, comprising a total fishing area of ca. 23,000 km^2^ (6,900 nm^2^). The equivalent of 4.3 full-time vessels operated year-round in the PTF between 2003 and 2009, with slightly reduced effort from December to March when cyclones are more frequent. The trawlers generally stay at sea for five to 12 days at a time, fishing throughout the day and night. Individual trawls ranged in duration from 30 min to five h, with a median trawl time of ca. 2.7 h. The total trawling effort ranged from 4,500 to 6,000 trawls (ca. 11,000 to 16,000 h) per annum (Table 1) between 2003 and 2009, though it has averaged ca. 9,000 h per annum from 2010 to 2012 [27].

**Table 1:** Total numbers of trawl days, hours and individual trawls in the PTF.

Year	Trawl days	Trawl hours	Trawl numbers
2003	1,014	14,663	1,107*
2004	953	15,372	5,591
2005	886	14,721	5,500
2006	914	15,792	5,882
2007	841	14,197	5,204
2008	831	11,966	4,533
2009	713	10,605	2,845*

*Not all trawl data for calendar year provided. These figures represent the subset subject to analyses, not the annual totals.

Numbers refer to those conducted by calendar year (January to December) in the Pilbara Trawl Fishery from 2003 until 2009 (source = Department of Fisheries [27]).

Data for this fishery are reported in three different 12-month intervals: The annual State of the Fisheries reports by the Department of Fisheries summarise data from January to December (calendar years); skippers' logbook data are summarised from July until the following June (Australian financial years) by industry for the Australian Taxation Office; and observer records for the fishery were summarised from October to September. In this study, we have presented data from the Department of Fisheries by calendar years for ease of comparison, but based our analyses of logbook and observer data on the industry format of the financial year (see Table 2).

**Table 2:** Dolphin bycatch rates reported by skippers and observers.

Period	Skippers' logbook			Independent observer		
	# dolphins	# trawls	#/1,000 trawls	# dolphins	# trawls	#/1,000 trawls
*a) No BRD*						
Aug03–Jun04	19	3,373	5.6	1	46	21.7
Jul04–Jun05	48	4,793	10.0	9	481	18.7
Jul05–Feb06	32	3,002	10.7	10	537	18.6
Total No BRD	99	11,168	8.9	20	1,064	18.8
*b) BRD*						
Jan05–Feb06	5	854	5.9	3	298	10.1
Mar06–Jun06	9	1,569	5.7	7	657	7.6
Jul06–Jun07	31	5,345	5.8	10	1,055	9.5
Jul07–May08	16	3,871	4.1	5	429	11.7
Total BRD	61	11,639	5.2	25	2,439	10.3
*c) BRD forward*						
Jun08–Jun09	18	4,365	4.1	7	621	11.3
Jul09–Sep09	2	732	2.7			
Total BRD forward	20	5,097	3.9	7	621	11.3
*d) BRD+BRD forward*						
Jan05–Sep09	81	16,736	4.8	32	3,060	10.5
**TOTAL**	**180**	**27,904**	**6.5**	**52**	**4,124**	**12.6**

Number of dolphins caught, number of trawls observed, and dolphin bycatch rate/1,000 trawls in Australian financial years of July to June (as per presented by industry in logbooks) and divided by net type (i.e. No BRD, after the introduction of BRDs, and then BRDs being moved forward in the extension).

**Figure 2:**
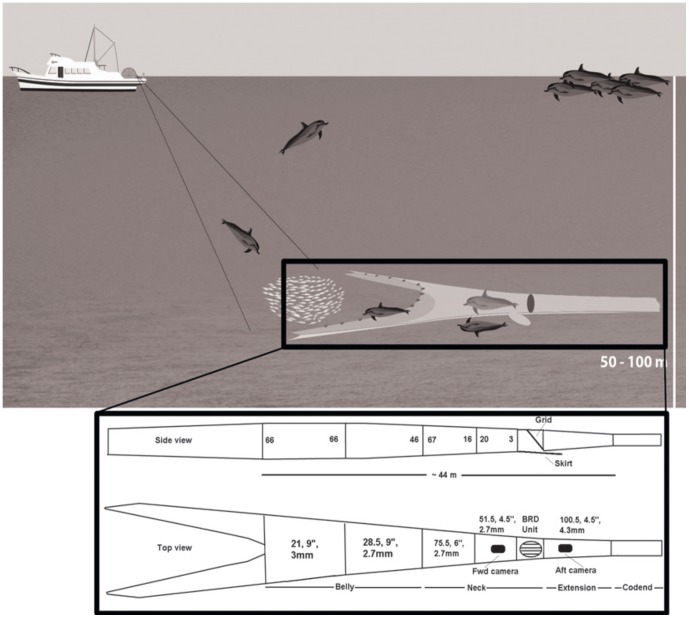
Schematic of trawler and trawl net on or near the seabed. Also illustrated are the typical positions of dolphins in and around the net, as well as following the trawler on the surface. The detailed net diagram represents the typical net specifications used in the Pilbara Trawl Fishery in 2008 and 2009, showing the side and top views, location of the Bycatch Reduction Device and the skirt covering the escape hatch. The lengths of the different panels are given as number of meshes, mesh length (in inches) and diameter of twine (in mm). In the side view, the height of each panel is given as the number of meshes. Diagram not to scale. Modified from Stephenson et al. [29] and Jaiteh et al. [35] following plans by H. McKenna for the ‘Magnet Box Diamond Net’ with short neck.

Trawl vessels in the PTF tow a single net at a speed of ca. three to three and a half knots (5.6–6.5 km/h), with twin otter boards maintaining the net spread (see also [35]). Most nets in the PTF consist of four main sections: the wings, which form the opening or mouth of the net; the belly and neck, which are immediately behind the mouth of the net and where the net tapers; the extension, a tubular section; and the codend, where the catch is collected (Fig. 2). The diameter and mesh size decrease in each panel with distance from the opening of the net. The length of the head rope must not exceed 36.6 m, while the total length of the net, including cables, sweeps and bridles, is limited to 274.3 m. The footrope is weighted and contains bobbins (<35 cm in diameter) that are spaced about 30 cm apart and roll along the sea floor.

Nets used in the PTF consist of diamond mesh. The first section of the net belly measures 4.8 m in length when the net is stretched. In October 2008, the belly and neck sections of the nets were shortened to allow for a shorter escape route for dolphins that enter the net and interact with the exclusion grid (Fig. 2). Based on stretch mesh measurements, the nets are approximately 44 m long from the footrope to the start of the codend and, when trawling/fishing, they are likely to be about 60–70% of this length.

Bycatch exclusion grids and escape hatches were trialled in 2004 and 2005, then fitted into all nets used in the PTF in March 2006 [29] [31]. The BRDs in use at the time of this study consisted of a semi-flexible metal grid and a bottom-opening escape hatch (through which large animals could leave the net), with a loose skirt of netting to prevent the loss of target species covering the hatch (see also [35]). The exclusion grid was held upright by a number of floats. The grid lay at an angle with the float-equipped top section anterior to the lower section, so that bycatch and benthos were deflected down toward the bottom-opening escape hatch. In June 2008, the BRDs were moved forward in the net, from just before the codend, to the start of the net extension. This was done to prevent dolphins from backing down into the extension and to provide a shorter escape route between the BRDs and the opening of the net. All grids featured vertical bars made of stainless tube and central sections of braided stainless wire.

The trawl data were categorised into three broad net types: 1) before the introduction of the BRDs (August 2003 until February 2006; excluding the BRD trials) – “No BRD”; 2) BRD trials from the previous period, after the compulsory introduction of the BRDs and before they were moved forward (primarily March 2006 to May 2008) – “BRD”; and 3) after the BRDs were moved forward in the net (June 2008 until September 2009) – “BRD forward”. The total number of trawls, number of dolphin bycatch events and dolphin catch rate per 1,000 trawls were calculated for each category (Table 2).

### Data analyses

The skippers' logbook and independent observer data from October 2003 to August 2009 for the PTF were provided by the Department of Fisheries and stored in a Microsoft Access database. The skippers' logbook data for this period of trawl fishing activity comprised information on targeted catch and bycatch from 30,684 trawls and the observer data set contained similar details from 4,939 trawls. Structured query language (SQL) queries were written to filter the dolphin bycatch data and location data were examined in ArcGIS. Summary figures and binary logistic generalised linear models were run in SPSS 16.01.

Excel files containing latitudes and longitudes of trawls were used to create point files in ArcGIS. The start and end latitudes and longitudes were combined using the Merge function and used to calculate the straight-line distance (line segment) of each trawl (in nautical miles, nm). Data were screened for trawls that were either outside the trawling management areas and/or line segments longer than 21 nm (39 km, equivalent to a trawl duration of about six to eight h). After removing these data, along with duplicate records and those with missing values, ca. 90% of the logbook data (or n∼11,200 trawls for the No BRD net type, ∼11,700 for the BRD net type and n∼5,100 trawls for the BRD forward category) and 85% of observer data remained for further analyses. The density of lines (trawls) was calculated using the Line Density function in ArcGIS.

As with net type, each of the trawl variables used in the analyses were placed into the following categories: Time of day (morning {06:00–11:59}/afternoon {12:00–17:59}/night {18:00–23:59}/early morning {0:00–05:59}); Area (1/2/4/5, fishery management area 3 is closed to trawling); Season (wet {December–April}/dry {May–November}); Vessel (1/2/3/4); Trawl duration (0.1–1.0 h/1.1–2.0 h/2.1–3.0 h/3.1–4.0+ h); Trawl distance (0.1–5.0 nm/5.1–10.0 nm/10.1–15.0 nm/15.1–20.0 nm); Trawl depth (51–60 m/61–70 m/71–80 m/81–90 m/91–100+ m); and dolphin bycatch (present/absent).

### Binary logistic generalised linear models

The categorical variables net type, time of day, management area, season, vessel and trawl duration were fitted as individual predictors for the presence of dolphin bycatch in separate binary logistic generalised linear models (GLMs) [36] for the logbook data and the observer data. These models were used to determine which variables were significant in predicting the presence of dolphin bycatch. The unit of measure for the presence of dolphin bycatch was the individual trawl and each is assumed to be independent. The significant predictors, and the interactions between them, were then used in combination in multi-predictor binary logistic GLMs. The multi-predictor GLMs were used to determine which combination of predictors accounted for the highest probability of the presence of dolphin bycatch. Akaike's Information Criterion (AIC) [37], which selects the most parsimonious model that best fits the data by taking into account the variation explained and the number of terms in the model, was used to select the best model. The lower the AIC value, the better the fit of the model [38].

Given the relatively low number of trawls in the observer data set for the BRD and BRD forward net categories, the data for these categories were pooled into a BRD category for No BRD versus BRD comparisons. For ease of comparison with earlier bycatch mitigation research by the Department of Fisheries [29] [31], the results of dolphin capture rates under the various conditions assessed are presented in dolphin captures per 1,000 trawls.

### Independent observer coverage levels

After the introduction of BRDs across the PTF in March 2006, scientific advice from within the Department of Fisheries suggested that minimum observer coverage from 2006–2007 onward should be at least 22% of total fishing effort and be representative of the operations of the fishery [29]. We calculated observer coverage rates in Australian financial years from the datasets provided. We also assessed what sample size of trawls should be monitored by observers to obtain an estimate of dolphin bycatch rates with a relative proportional standard error (Coefficient of Variation) of 20%. We used standard estimation equations for a population total based on simple random sampling from a finite population [39].

## Results

### Overall dolphin bycatch rates

The total number of trawls for a full calendar year in the PTF data provided was highest in 2006 (5,882 trawls; 15,792 h of trawling) and lowest in 2008 (4,533 trawls; 11,966 h of trawling). A total of 171 dolphin capture events, involving 180 dolphins, were recorded in the skippers' logbook data of 27,904 trawls from 2003 to 2009, at an overall rate of 6.5 dolphins/1,000 trawls (Table 2). Observers reported 48 dolphin capture events, involving 52 dolphins, in the observed subset of 4,124 trawls at an overall rate of 12.6 dolphins/1,000 trawls (Table 2). Note, however, that the dolphin bycatch rates varied among the broad categories of net type and were lower after Bycatch Reduction Devices (BRDs) were installed (Table 2, see also below).

In general, one dolphin was caught in the net, except on nine occasions in which skippers reported two dolphins caught in one trawl and four occasions in which observers reported two dolphins caught in one trawl. These data were too sparse to model actual counts, so the presence of a dolphin bycatch event (‘at least one dolphin caught’) was the measure used for the generalised linear models. Underwater video observations of dolphins in 44 operating trawls (ca. 1% of 2008–2009 effort) found that one or two dolphins typically swim inside the actively fishing trawl nets at a time, although up to nine dolphins have been recorded in the net at any one time [35].

### Spatial dolphin bycatch and fishing effort

The spatial distribution of lines, representing trawls from August 2003 until September 2009, indicated that fishing effort was most intense in Management Area 1 and least intense in the most remote (in terms of distance from home ports) northern and eastern regions of Management Area 5 (Fig. 1). The catch of dolphins appeared largely to reflect the intensity of fishing effort, with most dolphins captured in Area 1 (Fig. 1). From the logbook data, dolphin capture rates were greatest in Management Area 4, but for observer data, they were highest in Area 2. These differences in dolphin catch rates among areas were, however, not significant (Table 3).

**Table 3:** Presence of dolphin bycatch by individual factors.

Factor		Skippers' logbook			Independent observer		
	df	AIC	Likelihood ratio (χ^2^)	*P*	AIC	Likelihood ratio (χ^2^)	*P*
Time of day	3	29.07	44.03	<0.001	23.53	8.39	0.039
Net type (separate)	2	22.93	18.18	<0.001	19.25	5.18	0.075
Net type (pooled)	1	16.45	17.89	<0.001	13.87	5.06	0.025
Vessel	3	29.97	8.20	0.042	23.74	11.76	0.008
Trawl duration*	3	24.41	12.22	0.007	24.07	3.48	0.323
Trawl area	3	30.00	3.95	0.267	24.81	2.87	0.413
Season	1	16.46	0.01	0.904	13.87	0.34	0.853

*Trawl duration was estimated using a reduced dataset (AIC not directly comparable).

Results from binary logistic generalised linear models to predict the presence of dolphin bycatch in trawl nets by individual factors, based on skippers' logbook data and independent observer data (skippers' logbook number of trawls, n = 27,914; independent observer, n = 4,178, except for the predictor trawl duration, where logbook n = 27,489 and observer n = 4,153). df = degrees of freedom. Net type (separate) = analysis of three net types (No BRD, BRD, BRD forward). Net type (pooled) = analysis of two net types (data for BRD and BRD forward pooled).

### Predictors of dolphin bycatch

**Figure 3:**
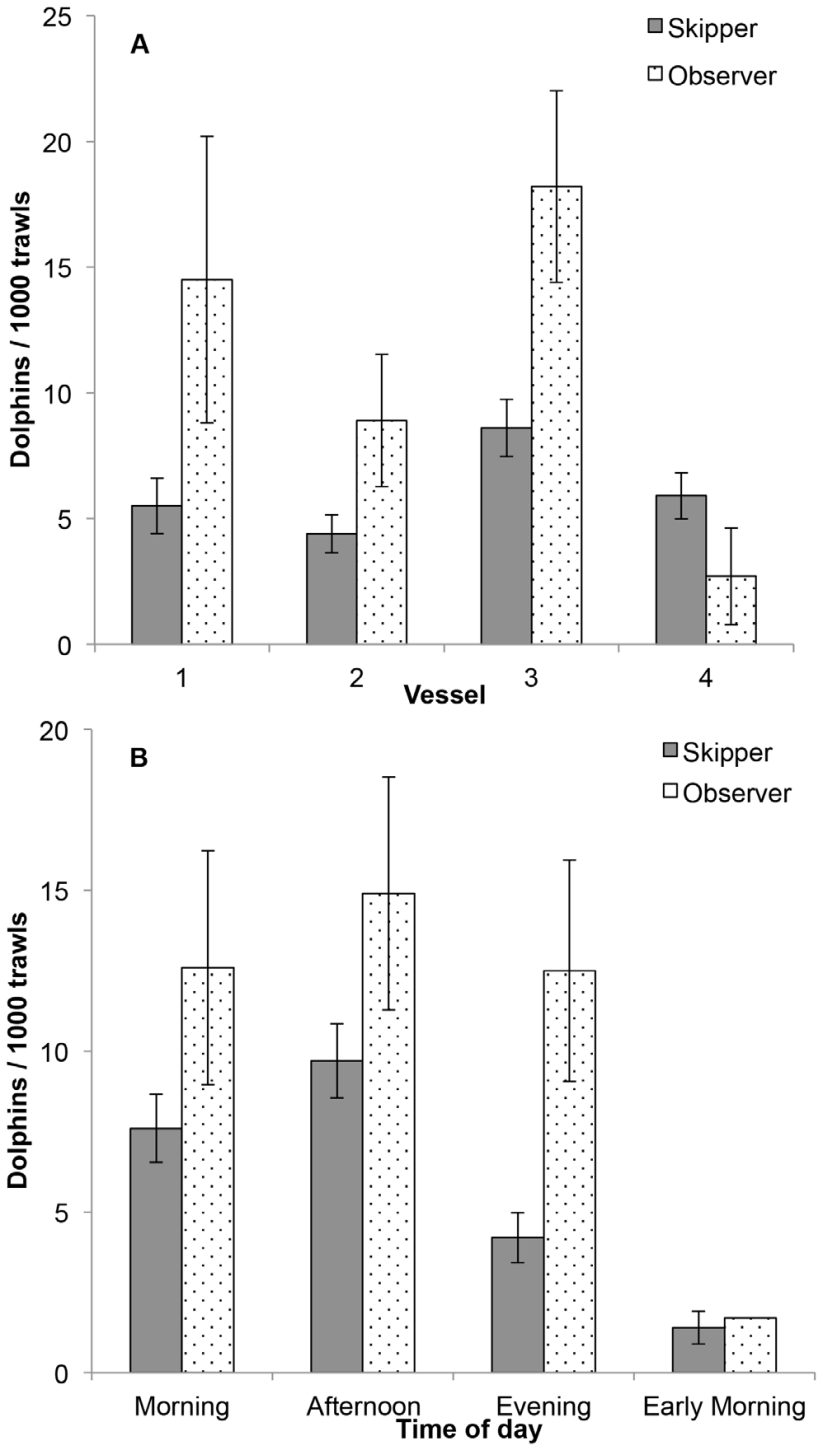
Dolphin bycatch rates by a) vessel and b) time of day. Mean (±1 SE) dolphin bycatch rates by a) vessel (1–4) and b) time of the day (Morning = 06:00–11:59; Afternoon = 12:00–17:59, Evening = 18:00–23:59; Early Morning = 00:00– 05:59) in the Pilbara Trawl Fishery. For skippers' logbook, n = 27,914; for independent observer, n = 4,178.

Binary logistic generalised linear models fitting single predictors found that vessel, net type (BRD and BRD forward pooled), and time of day were each significant in predicting the occurrence of dolphin bycatch in the PTF for both the skippers' logbook and observer data (Table 3). Trawl duration was also a significant predictor in the logbook data only (higher bycatch rates in longer trawls). In contrast, management area and season (wet versus dry) were not significant in predicting the occurrence of dolphin bycatch (Table 3). For the independent observer data, only vessel, time of day and net type (BRD and BRD forward pooled) were significant in predicting the occurrence of dolphin bycatch (Table 3). Rates of dolphin bycatch were significantly higher in one vessel than the other three and capture rates were significantly lower in the early morning than at other times of the day (Fig. 3a and b). While the magnitudes of dolphin capture rates were consistently higher for the observer than the logbook data, they followed a similar pattern of change for each factor (Fig. 3a and b, Table 3). The predicted dolphin capture rates did not differ significantly between different trawl durations, trawl distances, seasons or between depths (Table 3).

**Figure 4:**
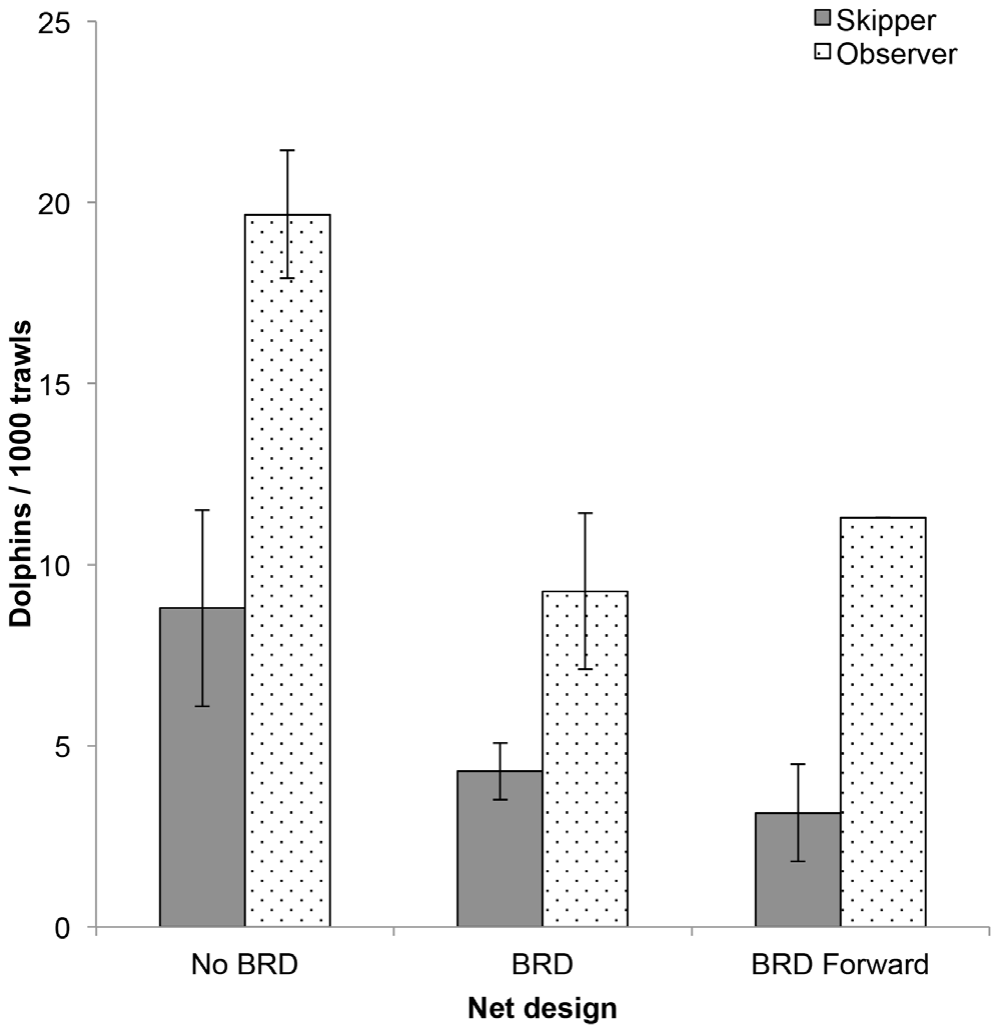
Dolphin bycatch rates by differing net design in the Pilbara Trawl Fishery. Mean (±1 SE) dolphin bycatch rates by differing net designs. BRD = BRD fitted just forward of the codend and at the aft end of the tubular net extension; BRD forward = BRD moved to the forward end of the extension.

The mean rates of dolphin bycatch differed markedly between skippers' logbooks and observer reports. Observer reported dolphin bycatch rates were 2.1, 2.0 and 2.9 times higher than those from logbooks in the No BRD, BRD and BRD forward periods of trawling activity, respectively (Table 2; Fig. 4). The number of observed trawls for the BRD forward category (n = 621) was much lower than in the prior two periods (n = 1,064 and 2,439, respectively). Observer reported catch rates were 2.2 times higher than the logbook reported rates in the BRD and BRD forward pooled category.

After the introduction of BRDs, the rate of dolphin bycatch from both the skipper and observer records declined by ca. 45% (Fig. 4). After the BRDs were moved forward in the nets, the logbook data showed a further slight decline in dolphin capture rates and the observer data indicated a slight increase in dolphin catch rates (Table 2; Fig. 4).

**Figure 5:**
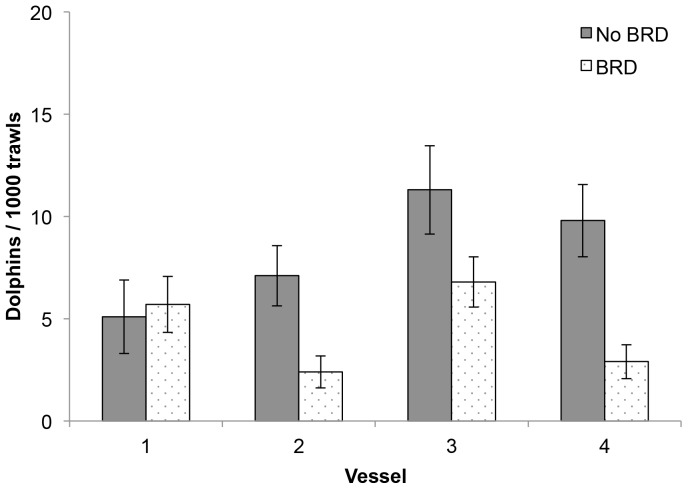
Dolphin bycatch rates net type x vessel interaction. Mean (±1 SE) dolphin bycatch rates illustrating the net type (No BRD vs BRD) x vessel (1-4) interaction based on skippers' logbook data.

From the skippers' logbook data, time of day, net type and vessel were significant in predicting dolphin bycatch, as was the net type×vessel interaction, although the effect was not nearly as strong as the main factors (Tables 4a and 5a). This interaction was due to the fact that dolphin capture events were lower in the BRD than No BRD net type category for three vessels, but remained the same for one vessel (Fig. 5). For the independent observer data, vessel and time of day were the strongest predictors of dolphin bycatch, while the net type effect was close to significance (*P* = 0.06, Tables 4b and 5b).

**Table 4:** Summary of full models to predict dolphin bycatch by fitting multiple factors for a) skippers' logbook data and b) independent observer data.

Full models	AIC	df	Model Log-likelihood	Likelihood ratio (χ^2^)	P
*a) Skippers' logbook data*					
*β*_0_+*β*_1_ *V*1+*β*_2_ *V*2+*β*_3_ *V*3+*β*_4_ *TOD*1+*β*_5_ *TOD*2+*β*_6_ *TOD*3+*β*_7_ *NT*1+*β*_8_(*V*1*NT1)+*β*_9_(*V*2*NT1)+*β*_10_(*V*3*NT1)	130.16	10	−54.08	82.63	<0.001
*b) Independent observer data*					
*β*_0_+*β*_1_ *V*1+*β*_2_ *V*2+*β*_3_ *V*3+*β*_4_ *TOD*1+*β*_5_ *TOD*2+*β*_6_ *TOD*3+*β*_7_ *NT*1	87.81	7	−35.89	24.27	0.001

Results from binary logistic generalised linear models for predicting the occurrence of dolphin bycatch in the Pilbara Trawl fishery using time of day, net type, vessel and the net type×vessel interaction as predictors from a) skippers' logbook data (number of trawls, n = 27,914) and b) observer data (n = 4,178).

**Table 5:** Model comparison with the full model (Table 4) for predicting dolphin bycatch by fitting multiple predictors for a) skippers' logbook data and b) independent observer data.

Predictors	df	Likelihood ratio (χ^2^)	*P*
*a) Skippers' logbook data*			
Intercept	1	33,323.66	<0.001
Time of day (*TOD*)	3	46.99	<0.001
Net type (*NT*)	1	15.15	<0.001
Vessel (*V*)	3	12.58	0.006
Net type * Vessel	3	8.57	0.035
*b) Independent observer data*			
Intercept	1	3,375.33	<0.001
Vessel (*V*)	3	11.05	0.011
Time of day (*TOD*)	3	8.89	0.031
Net type (*NT*)	1	3.49	0.062

Model comparison compares the full model (from Table 4) with the reduced model to indicate the significance of the additional predictors from a) skippers' logbook data (number of trawls, n = 27,914) and b) observer data (n = 4,178).

### Independent observer coverage levels

From the 2003–2009 data provided for this study, the overall observer coverage was ca. 16%. Observer coverage levels attained after the 22% minimum was recommended by internal Department of Fisheries scientific advice were 17% in 2006–2007, 13% in 2007–2008, 13% in 2008–2009 and ca. 8% from July to September 2009.

Given a “population” of 3,000 trawls (six-eight months of trawling in the PTF), with a dolphin bycatch rate of 20 dolphins/1,000 trawls (roughly equivalent to the rate reported by observers prior to the introduction of BRDs), about 30% observer coverage would be required in order to estimate bycatch rates with a relative proportional standard error of 20%. For dolphin bycatch rates of 10 dolphins/1,000 trawls (approximating the rate reported by observers after the introduction of BRDs), 46% of the 3,000 trawls would need to be monitored and, for five dolphins/1,000 trawls (the rate reported by skippers after the introduction of BRDs), 62% of the 3,000 trawls would need to be observed.

## Discussion

In this study, we evaluated detailed, long-term catch and effort data from the Pilbara Trawl Fishery to assess patterns of dolphin bycatch and determine whether the introduction of spatial and/or temporal fishery management measures might contribute to mitigating dolphin bycatch. We also aimed to quantify differences in dolphin bycatch rates by net type. In general, the trends in dolphin bycatch rates from skippers' logbooks and independent observer datasets followed similar patterns of variation with vessel, time of day and the net type (with or without Bycatch Reduction Device) in use. The reported rates of dolphin capture from the logbooks were consistently lower (<½) than those reported by observers. This is consistent with the reporting of bycatch in a number of other fisheries; particularly where the capture of marine mammals is illegal [5] [40]. Assuming the number of dolphin capture events reported in the 2010–2012 logbooks [27] [41] was ca. ½ the number observers would have reported, had they continued after 2009, a minimum of ca. 500 bottlenose dolphins were caught in the PTF in the decade 2003–2012.

The analysis of dolphin bycatch patterns on spatial, daily and seasonal scales, and comparisons among different net designs, contradicted some of our expectations. For example, the greatest source of variation was not the net type (No BRD vs BRD/BRD forward). Most of the variation in dolphin bycatch was explained by the predictor variables of vessel and time of day. In the full model, net type was significant for the skippers' data and close to significance for the observer data. While the logbook data was a much larger dataset (ca. seven times more trawls than the observer dataset), the observer data better explained variation in dolphin bycatch, perhaps because skippers missed some bycatch events (i.e. when dolphins fell out of the bottom-opening escape hatch on winch-up and prior to being landed on deck). Accordingly, most of the discussion focuses on the results from the observer data.

### Temporal patterns of dolphin bycatch and fishing effort

Observer reported dolphin catch rates in the six-hour period of early morning (00:00–05:59), when the least fishing occurs, were up to 85% lower than in the other three 6 h periods of the day. Logbook records also indicated a similar pattern, although the difference between periods was not as marked as those from the observer data. This result is reasonably consistent with the smaller subset of data summarised by Stephenson and Wells [31]. Bycatch records collected between January 2004 and June 2005 indicated that 92% of dolphins were caught between 7am and 8pm [31]. It is difficult to determine why dolphins might be less likely to be caught in the late evening and early morning. Bottlenose dolphins were seen foraging around the trawlers in the PTF throughout the day and night (SA, pers. obs.), though it was not possible to determine if this occurred to the same extent at night as it did during the day. Bottlenose dolphins (*Tursiops* spp.) also foraged around trawlers throughout the day and night in Moreton Bay, Queensland [42], and Spencer Gulf, South Australia [43]. In a study of two fisheries off the north-eastern United States, Waring et al. [44] noted that the bycatch of common dolphins (*Delphinus* sp.) and pilot whales (*Globicephala* sp.) tended to follow a diel pattern, with common dolphins being caught at night and pilot whales caught during the day.

Bottlenose dolphins interacting with trawlers in the PTF may have adopted a diurnal pattern of behavior in response to this foraging association, just as the behavioral budgets and social structure of bottlenose dolphins have adapted to other circumstances in which anthropogenic activities mediate a particular schedule or regimen [45]. Numerous studies have demonstrated the adaptability of bottlenose dolphin behavior to human activity, for example: dolphins spent less time in Milford Sound, New Zealand, during periods of intense tour boat activity [46]; free-ranging dolphins adopt a daily activity pattern to take advantage of provisioning by tourists in Shark Bay, Western Australia [47]; and artisanal fishing in Laguna, Brazil, and trawl fishing in Moreton Bay, Queensland, have documented influences on the activity budgets and association patterns of bottlenose dolphins [42] [48] [49]. This rapid learning ability and behavioral flexibility means that the chances of dolphins interacting with boats and fishing gear are greatly increased, particularly when food is an incentive [50].

Underwater video data collected in daylight hours suggests that dolphins spend considerable time foraging in and around trawl nets in the PTF [35]. If this foraging effort is sufficient to procure their daily food requirements, the dolphins may be less inclined to do so late at night and in the early morning, opting instead to forage less and remain vigilant against predation. Known predators of dolphins, such as sandbar (*Carcharhinus plumbeus*), oceanic white tip (*C. longimanus*) and tiger (*Galeocerdo cuvier*) sharks, also follow trawlers in the PTF (SA, pers. obs.). Dolphins have been shown to modify their habitat use in response to the presence of tiger sharks [51]. Regardless of the reasons behind the lower bycatch rate in the early mornings, a restriction of fishing activity in the daytime and concomitant increase at night would be unlikely to reduce dolphin bycatch rates in the medium- to long-term, as the dolphins may be able to adapt their behavior to this change in fishing activity.

Both skippers' logbook and independent observer data suggested that season had little influence on the likelihood of dolphin bycatch in the PTF. Although relatively little is known of the ecology and movements of bottlenose dolphins interacting with the PTF (but see [35]) or, indeed, those in any of Australia's extensive pelagic waters [21], this lack of an effect is not surprising. Prevailing winds and rainfall levels do change on a seasonal basis in this region, but there are no marked changes in the physical or biological conditions (such as water temperature or prey abundance/density) in the pelagic environment that might be expected to result in seasonal fluctuations in the numbers of dolphins in the area. Furthermore, underwater video footage, photographic identification and genetic evidence indicates that at least some individual dolphins show fidelity to foraging around trawlers for periods of weeks to years [32] [35].

### Vessel and net type effects on the probability of dolphin bycatch

A strong vessel effect was evident in both the logbook and observer data, and in both single- and multi-predictor generalised linear models (GLMs). One vessel had higher bycatch rates than the other three assessed. The difference in dolphin catch rates among vessels is difficult to interpret, especially given the similarities in boat configurations and nets in this small fishery. It may be attributable to the different fishing practises employed by different skippers in the fleet. For example, some skippers tend to conduct their fishing operations in a very consistent manner over time, while others tend to modify how they are fishing on a frequent basis. Furthermore, some skippers tend to take more risks than others in terms of trawling over or near benthic structures, such as rocky reefs or pipelines associated with the offshore oil and gas industry. It is likely that more consistent trawling, and therefore fewer instances of rapid winch-up or net collapse, results in fewer dolphin captures.

Net type (No BRD vs BRD vs BRD forward) was also significant in predicting dolphin bycatch in the single and multi-predictor GLMs using logbook data. In the observer data, net type (No BRD vs BRD/BRD forward pooled) was significant in the individual model, and close to significance in the multi-predictor model. The relatively small sample size of observer coverage for the BRD forward design, along with the relatively low incidence of dolphin capture, reduced the power to detect any change/effect among the three net designs in the observer data. Differences in skipper behavior and detail of reporting dolphin bycatch are likely to account for the interaction detected between vessel and net type detected in the skippers' logbook dataset.

Both the skipper and observer reported rates of dolphin bycatch dropped by ca. 45% after the introduction of BRDs, consistent with the earlier assessment of the smaller subset of data [29]. The trends after the forward movement of the BRDs, however, were inconsistent between skipper (a slight further decrease in dolphin catch rates) and observer data (a slight increase) and no significant changes were detected in either dataset. Exclusion grids and escape hatches of various forms have been trialled to reduce bycatch of marine mammals, turtles and other megafauna in numerous trawl fisheries around Australia and the world. While detailed measures of their efficacy, including long-term follow-up, are scarce, those that have met with some success include the following: Northridge et al. [52] have experimented with exclusion grids and top-opening escape hatches in an English pelagic bass pair-trawl fishery, reporting reductions in common dolphin bycatch without the loss of target species; Zeeberg et al. [53] report on the use of escape hatches to reduce the bycatch of a number of species of small cetaceans and other megafauna in the Dutch trawl fleet fishing off Mauritania; top-opening escape hatches and exclusion grids have reduced the bycatch of turtles, large sharks and rays in Australia's Northern Prawn Fishery [54]; the bycatch and mortality rates of fur seals (*Arctocephalus* spp.) were reduced with the use of large, bottom-opening escape hatches in a pelagic, mid-water trawl fishery off Tasmania [55].

Since the reported reduction in dolphin bycatch rates in the PTF after the introduction of BRDs in early 2006 (this study, [29]), annual fishing effort has declined by ca. 35% (2006 = 15,792 h; 2012 = 10,269 h) [27]. According to the latest skippers' logbook data, however, dolphin bycatch rates have increased above those reported immediately after the BRDs were made mandatory (2006 = 2.2 dolphins/1000 h trawling; 2012 = 2.8 dolphins/1000 h trawling) [27]. This minimum estimate, combined with underwater video footage showing a proportion of incidentally caught megafauna falling out of the bottom-opening escape hatch before being landed on deck [32], indicates that bycatch rates reported by both skippers and observers are invariably under-estimates and that BRDs are unlikely to be as effective as first presumed. Similarly, in South Australia, an unknown proportion of endangered Australian sea lions (*Neophoca cinerea*) sustain life-threatening injuries or die in gill nets and drop out before being detected, even by vigilant onboard observers [56] [57]. Furthermore, bottom-opening escape hatches are not well suited to dolphins and other air-breathing animals in the PTF, which tend to swim upward and push on the upper ceiling of the net (in an attempt to get to the surface) when trying to escape [32].

### Spatial patterns of dolphin bycatch and fishing effort

Logbook data suggested that dolphin capture rates were highest in Management Area 4 of the PTF, while observer data indicated the highest rate was in Area 2. These differences were not significant in predicting dolphin capture in either single- or multi-predictor GLMs based on observer data. Nor were there any marked differences in capture rates by depth in the logbook or observer data. These results, based on six years of data, corroborate the earlier study by Stephenson and Wells [31] from an 18-month subset of data and are to be expected, due to the broad extent of interactions between dolphin and trawlers operating in the PTF in both space and time [32] [35]. Dolphin bycatch events are spread across the four Management Areas open to trawling and across all depths (50–100+ m) in the fishery. Fernández-Contreras et al. [58] suggested that limiting trawling to deeper waters would reduce common dolphin bycatch in the pelagic trawl fishery off north-western Spain. The operators in the PTF have periodically undertaken spatial restrictions in fishing effort in response to declining stocks of target species in the past [27], but the results of our study suggest that such a spatial restriction within the PTF would be highly unlikely to reduce dolphin bycatch.

### Skippers' logbook data and independent observer coverage

The extensive databases of logbook and observer records formed the basis of this assessment of spatial and temporal patterns of dolphin bycatch in the PTF, but some problems were evident in the quality of the data. Due to errors such as blank fields and erroneous location data in the logbook dataset, only ca. 90% of the trawl records could be analysed. More blank fields and errors were seen in the observer data, particularly from 2004 to 2006, and only ca. 85% of these records were of sufficient quality for analyses. Sound reporting practices and validation checks would improve the quality of these data sources and their value for interpreting patterns of bycatch.

The dolphin bycatch rates from the independent observer coverage data were over double those reported in the skippers' logbooks. The under reporting of bycatch by skippers is not unusual [5] [40], but highlights the importance of having enough observer coverage to provide robust estimates of dolphin bycatch and other incidental catches in non-selective fisheries. The Department of Fisheries specified that minimum observer coverage of 22% of total fishing effort was required from 2006–2007 onward [29], but the 2003–2009 data indicated that overall coverage was ca. 16% and declined over time from 17% in 2006–2007 to 8% in late 2009, when it ceased. This low and declining coverage, combined with the relatively infrequent incidence of dolphin capture, means that estimates of dolphin bycatch rates are imprecise and that the comparisons of dolphin bycatch rates between the different net designs have low power [32].

We calculated the amount of observer coverage required at between 30% and 62% of total fishing effort. Due to the high financial cost of independent observer programs, an electronic observer system involving deck-mounted video cameras was trialled in the PTF as an alternative to human observers [59]. The evaluation of this system concluded that it should not be used to replace independent observers, because of: the technical difficulties associated with maintaining the system in such a remote fishery; the system's lack of capability in differentiating between species of both targeted catch and incidental bycatch; the system's inability to detect dolphins that are caught and then fall from the BRD's escape hatch prior to being landed on deck; and the fact that the system is only of moderately lower cost than human observers when compared over a five-year period with observer coverage rates of ca. 60% or more [59]. The cost advantage of an electronic observer system should also be considered against the human observer's better reliability (far lower chance of data loss), ability to collect more detailed and accurate data on both target and non-target catch and capability to perform other tasks (such as otolith collection) for research and management purposes [59]. The independent observer program has not recommenced since its cessation in September 2009 and the Department of Fisheries adopted an electronic observer system for further trials in the PTF in 2012. Thus, more recent comparisons of the dolphin bycatch rates reported by skippers and independent observers cannot be made.

### Acoustic pingers as an alternative strategy for mitigating dolphin bycatch

Acoustic alarms or deterrents, “pingers”, were designed to alert marine mammals to the presence of fishing gear and/or deter them from approaching fishing gear and aquaculture operations. They are often deployed in static fisheries, such as gill nets and long lines. The Department of Fisheries conducted trials of trawl nets equipped with active and inactive pingers and monitored by underwater video cameras in the PTF, yielding no differences in the number of dolphins swimming into the nets [31]. Pinger trials were subsequently abandoned in favour of the compulsory introduction of BRDs across the fishery [29]. The Department of Fisheries have subsequently (2012) commenced another trial of larger, louder pingers in the PTF [27], but results are yet to be reported. Pingers have been shown to reduce the bycatch of some cetaceans, including harbor porpoises (*P. phocoena*), Franciscana dolphins (*Pontoporia blainvillei*) and common dolphins (reviewed in [30]). However, they do not elicit consistent responses in all small cetacean species, nor do they have the same affects across all types of fisheries. For example: gill nets equipped with active pingers induced only subtle behavioral changes, rather than an avoidance response, in bottlenose dolphins [60]; a more recent study found fewer bottlenose dolphins approaching within 100 m of pinger-equipped gill nets, suggesting that pingers reduce the frequency of, but do not eliminate, interactions [61]; and Berg Soto et al. [62] found that pingers elicited only subtle behavioral responses in Australian snubfin and Indo-Pacific humpback dolphins, suggesting they may not be effective in reducing bycatch of these species in gill nets or anti-shark meshing for bather protection and that alternative mitigation measures should be explored.

Pingers deployed in a pelagic pair trawl fishery did not reduce common dolphin bycatch [63], although more recent trials of louder pingers showed some promise [64]. The sample sizes used in the recent trials were, however, too small to provide statistically robust evidence of their efficacy [64]. Entanglements of bottlenose dolphins in various pinger-equipped fishing nets suggest that they are not an effective means of bycatch mitigation for this species [30]. The bottlenose dolphins interacting with the PTF exhibit a number of attributes that suggest that pingers are unlikely to deter them from interacting with the trawl nets or to reduce dolphin bycatch: for example, bottlenose dolphins are known to be behaviorally flexible [45]; they are not only aware of the presence of trawl fishing gear, but highly motivated by foraging and socializing opportunities to interact with the gear [35]; and, they appear to show fidelity to the region and foraging around trawlers [32] [35].

### Conclusions and recommendations

1. A minimum of ca. 500 bottlenose dolphins were incidentally caught in the Pilbara Trawl Fishery in the last decade;
2. Spatial and/or temporal fisheries management adjustments to fishing effort are unlikely to be effective in significantly reducing dolphin bycatch, as the extent of interactions between the dolphins and the PTF are great, the motivations for the dolphins to interact with the PTF and undertake risky behavior are considerable and bottlenose dolphins are behaviorally adaptable;
3. Pingers are unlikely to be effective in reducing the level of interactions between bottlenose dolphins and the PTF, due to the active and already-noisy nature of trawl fisheries (i.e. dolphins are aware of the presence of the fishing gear irrespective of the pingers), as well as the reasons listed above regarding dolphin behavior;
4. There has been no further reduction in dolphin bycatch since the Bycatch Reduction Devices were introduced, with an unknown quantity of bycatch falling out of bottom-opening escape hatches and, thus, not being reported;
5. Modified BRDs, with top-opening escape hatches, may be a more effective means of reducing dolphin bycatch;
6. Extensive independent observer coverage, as well as in-net video collection, are essential in order to quantify bycatch and estimate any reductions in bycatch with greater precision and statistical power following modifications to BRDs;
7. Data on dolphin population size and connectivity with adjacent populations are essential in order to calculate acceptable levels of human-caused dolphin mortality and determine the effectiveness of modified BRDs.

## Supporting Information

File S1 **Subsurface behaviour of dolphins in the Pilbara trawl fishery (Jaiteh et al 2013) Marine Mammal Science.**
(PDF)
