# Supplementary material for: Patterns of Dolphin Bycatch in a North-Western Australian Trawl Fishery
Source: PLoS One. 2014 Apr 2;9(4):e93178. doi: 10.1371/journal.pone.0093178 (PMC3973695; doi:10.1371/journal.pone.0093178)
Supplement: File S1 — Subsurface behaviour of dolphins in the Pilbara trawl fishery (Jaiteh et al 2013) Marine Mammal Science. (PDF) [file pone.0093178.s001.pdf]

Marine Mammal Science

MARINE MAMMAL SCIENCE, **(*): ***–*** (*** 2012)
© 2012 by the Society for Marine Mammalogy
DOI: 10.1111/j.1748-7692.2012.00620.x

Subsurface behavior of bottlenose dolphins (*Tursiops truncatus*) interacting with fish trawl nets in northwestern Australia: Implications for bycatch mitigation

VANESSA F. JAITEH[1] and SIMON J. ALLEN, Murdoch University Cetacean Research Unit, Centre for Fish, Fisheries and Aquatic Ecosystems Research, School of Biological Sciences and Biotechnology, Murdoch University, Murdoch, Western Australia 6150, Australia; JESSICA J. MEEUWIG, Centre for Marine Futures and School of Animal Biology, Oceans Institute, University of Western Australia, Crawley, Western Australia 6009, Australia; NEIL R. LONERAGAN, Murdoch University Cetacean Research Unit, Centre for Fish, Fisheries and Aquatic Ecosystems Research, School of Biological Sciences and Biotechnology, Murdoch University, Murdoch, Western Australia 6150, Australia.

ABSTRACT

Most studies of delphinid-trawler interactions have documented the surface behavior of dolphins feeding on discarded bycatch, but not their subsurface behavior around demersal trawl gear. Using video cameras mounted inside trawl nets, we recorded the subsurface behavior of common bottlenose dolphins (*Tursiops truncatus*) in a demersal fish trawl fishery in northwestern Australia. Footage from 36 trawls across the fishery was analyzed to determine the extent of dolphin-gear interactions and the behavior of dolphins inside the nets. Interaction rates were high, with dolphins present inside and outside the nets during 29 and 34 trawls, respectively, and for up to 99% of the trawl duration. The proportion of foraging behaviors exhibited inside the nets was higher than the proportions of traveling and socializing behaviors. Twenty-nine individuals were identified inside the net, seven of which returned repeatedly within and between trawls and fishing trips, but were observed primarily in the same localized areas in which they were first recorded. Our results suggest that entering trawl nets may be a frequently occurring, yet specialized behavior exhibited by a small subset of trawler-associated dolphins. We propose that gear modifications, not spatial or temporal adjustments to fishing effort, have the greatest potential to reduce dolphin bycatch.

Key words: bottlenose dolphin, *Tursiops truncatus*, underwater video observation, protected species, bycatch reduction.

Dolphins are apex predators whose movement patterns, like those of fishing vessels, are largely determined by the availability of prey (Shane *et al.* 1986). This often results in considerable overlap in the spatial distribution of fishing vessels and delphinid populations (Nitta and Henderson 1993). Due to their remarkable flexibility in foraging strategies, many delphinid communities have learned to exploit fisheries as

[1]Corresponding author (e-mail: v.jaiteh@murdoch.edu.au).

an energetically efficient food source, since fish are concentrated by the fishing activity and the energy expended on foraging can be lower than under natural conditions (Shane *et al.* 1986, Fertl and Leatherwood 1997). Delphinid interactions with fishing gear have been documented most thoroughly in gill net fisheries (*e.g.*, Dawson *et al.* 2001, Read *et al.* 2003, Rojas-Bracho *et al.* 2006, Bearzi *et al.* 2008), but are also known to occur in other fisheries, including long lines (*e.g.,* Dalla Rosa and Secchi 2007), drift nets (*e.g.,* Rogan and Mackey 2007), purse seines (*e.g.,* Hall 1998), fish and prawn trawlers (*e.g.,* Waring *et al.* 1990, Couperus 1997, Broadhurst 1998) and fish cage aquaculture (*e.g.,* Diaz-Lopez *et al.* 2005). While the term "interaction" has been used with a broad range of definitions in the literature, here it is defined as any association with, or close proximity to, the trawl net while it is actively fishing and does not indicate a bycatch event, *i.e.,* the incidental capture of a dolphin.

Associations between dolphins and trawlers are known from around the world (Waring *et al.* 1990, Fertl and Leatherwood 1997, Zeeberg *et al.* 2006, Gonzalvo *et al.* 2008), including various locations around Australia (Corkeron *et al.* 1990, Hill and Wassenberg 1990, Broadhurst 1998, Svane 2005). While these interactions provide dolphins with foraging opportunities, they also present risks of injury and mortality through entanglement in fishing gear. Fishing-related mortality is considered the most severe and immediate threat to populations of small cetaceans worldwide (Read 2008).

The Pilbara Fish Trawl Interim Managed Fishery (hereafter the "Pilbara Trawl Fishery" [PTF]) operates off the northern coast of Western Australia. Common bottlenose dolphins *Tursiops truncatus* (hereafter "bottlenose dolphins"), listed as a protected species in Australia, have been interacting with the PTF in the last decade, leading to a reported annual bycatch of 17–50 dolphins, depending on whether data recorded in skippers' logbooks or by observers are used to estimate the annual rate of bycatch (Allen and Loneragan 2010, Department of Fisheries 2011). Groups of dolphins follow the trawlers for extended periods of at least several days (Allen and Loneragan 2010), which suggests a close and ongoing interaction between the dolphins and the fishery, similar to that reported from Moreton Bay, Queensland (Chilvers and Corkeron 2001).

Virtually all previously published studies of dolphin-trawler interactions have been based on surface observations, stomach content analyses of incidentally caught dolphins, or examination of the composition and condition of the catch and gear once they are landed on deck (*e.g.,* Couperus 1997, Gonzalvo *et al.* 2008, but see Broadhurst 1998, who described subsurface interactions between bottlenose dolphins and the cod end of prawn trawl nets). Interactions between small cetaceans and fishing operations are generally assumed to originate from the animals' attraction to an easily accessible, concentrated food source, such as discards or the large numbers of prey around the nets (Hill and Wassenberg 1990, Fertl and Leatherwood 1997, Svane 2005).

In addition to feeding on discards from trawl catches, dolphins are known to interact with actively fishing trawl nets in the PTF. A recent investigation found that dolphins entered the nets in 66% of all trawls and were present inside the nets for up to 64% of the duration of each trawl (Mackay 2011). However, the video cameras used in that study were not able to record the entire duration of each trawl and did not capture footage of sufficient quality to identify individual dolphins in the net. Here, we used more advanced underwater video systems that were able to record the entire duration of the trawls and had the resolution to allow individual dolphins to be identified, to study the fine-scale nature of interactions between bottlenose dolphins and

actively fishing trawl nets. We also documented the extent of individual dolphin resightings inside the nets to assess whether entering trawl nets was restricted to a subset of individuals within the community of dolphins that associate with trawlers in the fishery. Data from this study provide an improved understanding of dolphin subsurface behaviors and further information for assessing the extent of dolphin-trawler interactions.

Materials and Methods

Study Site and Trawl Nets

The PTF operates between the 50 m and 100 m depth contours seaward of the Pilbara region in northwestern Australia, north of 21º44'S and between 114º9'36"E and 120ºE (Fig. 1). Areas 1, 2, 4, and 5 in the PTF are currently open to trawling, covering 6,900 nmi^2 (12,779 km^2) (Fig. 1). Three to four trawlers fish the area throughout the year, with reduced effort during the cyclone season from December to March. Fishing time is capped for each area of the fishery, with total annual effort across the entire fishery varying between *ca.* 11,000 and 16,000 h (*ca.* 4,500 and 5,500 trawls) from 2003 to 2009. In 2008, fishing effort in the PTF was 11,966 h (Department of Fisheries 2011). Individual trawls last approximately 3 h, but may be shorter or longer depending on fishing conditions (Allen and Loneragan 2010,

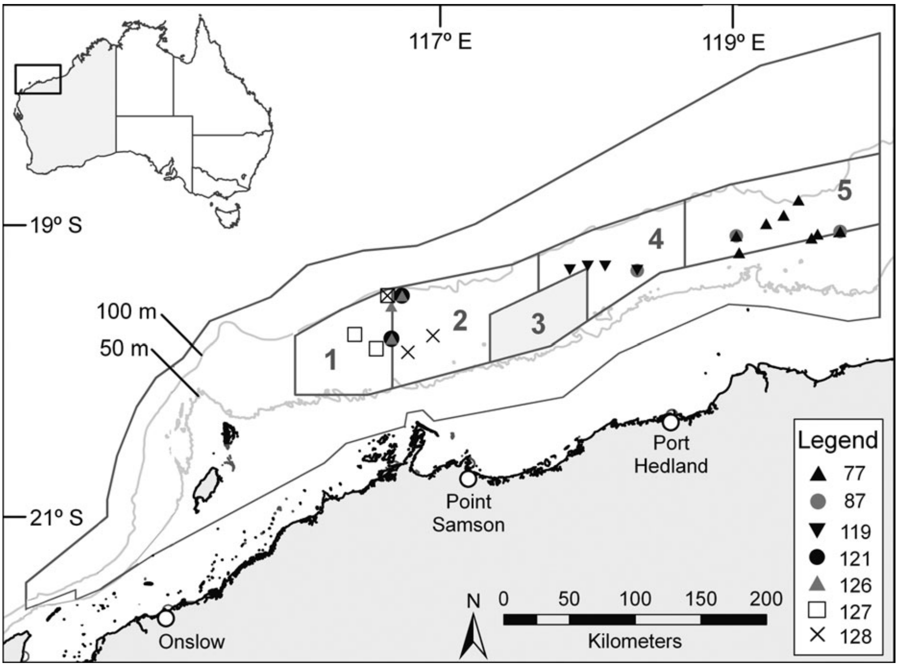

Figure 1. Map showing the location of the Pilbara Fish Trawl Interim Managed Fishery (PTF) in northwestern Australia and the position of the seven dolphins resighted in different fishing trips. Symbols correspond to ID numbers of individual dolphins (see legend). Areas 1, 2, 4, and 5 are open to trawling.

Department of Fisheries 2011). The PTF targets demersal finfish, including various snapper and emperor species (*Lutjanus* spp. and *Lethrinus* spp.) and Rankin cod (*Epinephelus multinotatus*) (Newman *et al.* 2003). It is mandatory for skippers in the PTF to report incidental catches of threatened and protected species, including dolphins, sharks, rays, turtles, sea snakes, sea horses, and pipefish, to the Commonwealth Government.

Trawl vessels in the PTF tow a single net with twin otter boards along the sea floor at a speed of just over three knots. The nets are divided into four main sections: the wings, which form the opening of the net; the throat, which is the panel immediately behind the opening of the net and where the net tapers, leading to a Bycatch Reduction Device (BRD) consisting of a bycatch exclusion grid and a downward-opening escape hatch covered by a net skirt; the extension, a tubular section; and the codend,

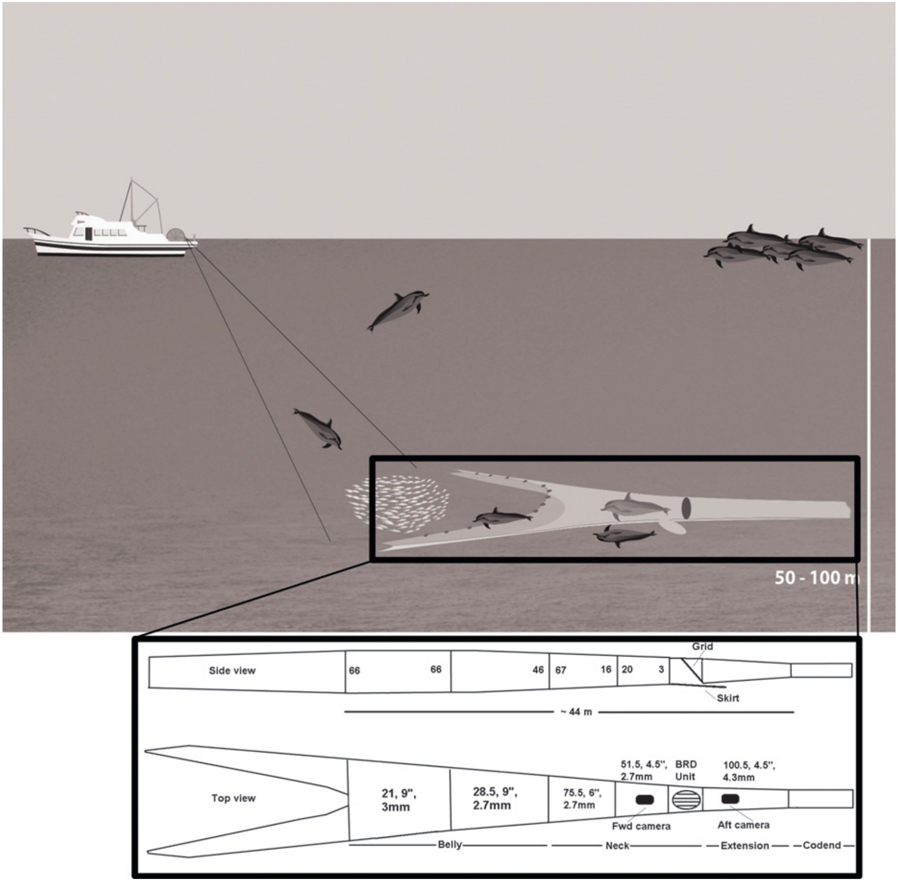

Figure 2. Graphic showing the position of an active trawl net on or near the seafloor, direction of net opening, and typical positions of dolphins in and near the net/following the trawler on the surface. Detailed net diagram shows net specifications and Bycatch Reduction Device (BRD) unit with downward-opening escape hatch covered by a skirt of netting and position of the forward (fwd) and rear (aft) video cameras. Net diagram modified from Stephenson *et al.* (2006) following net plans by H. McKenna. Figure not drawn to scale.

where the catch is collected (Fig. 2). The diameter and mesh size decrease in each panel with distance from the opening of the net; the minimum mesh size is 100 mm. Nets in the PTF typically have an opening of 15 m in height and a length of approximately 44 m from the end of the wings to the end of the extension. The codend often varies in length between different nets. The foot rope is weighted and contains bobbins (<35 cm in diameter) that are spaced about 30 cm apart and roll along the sea floor.

Data Collection and Video Analyses

A total of 85 h of video footage from 36 trawls was analyzed in this study. These data were collected in October and November 2008 by independent observers onboard a trawl vessel from the PTF during its normal trawling operations (Allen and Loneragan 2010). Trawling occurs throughout the day and night, with slightly reduced effort at night. The 36 daytime trawls that were analyzed for dolphin presence/absence and behavior inside trawl nets were completed during three fishing trips of approximately two weeks duration each, in all open areas of the fishery (*i.e.*, Areas 1, 2, 4, and 5, Fig. 1). During these trips, observers also made approximate counts of dolphins surrounding the vessel while the net was winched up.

Underwater video recordings were made during commercial fishing activities, using high definition Sony video cameras (HDR-CX7). The cameras were placed in waterproof metal housings and secured to the trawl net. A trawl net float was attached to the base plate of the housing and the netting behind the unit to compensate for the weight of the housings. A camera was fitted in the throat of the net, 3.6 m forward of the exclusion grid and facing forward toward the net opening (Fig. 2). Cameras were set to standard definition, long play and night vision to provide recording times in excess of 3 h and clearer recordings at depths where natural light was limited.

The video footage was viewed and analyzed using EventMeasure v2.04, a software package designed to record biological and behavioral information about animals in underwater movie sequences (Seager 2008). This program features an integrated movie player that supports efficient video analysis through fast forward playback and frame stepping functions. Events are logged by overlaying dot points on still images, with the identified individual marked by a red dot. Information and attribute fields can be loaded from a predefined text file and assigned to the overlaid points. At the end of a video sequence, the data added to the information and attribute fields can be exported as a text file for subsequent analyses. Furthermore, reference images and movie clips can be captured and recalled through an inbuilt viewer while analyzing video sequences. This function allows individuals to be identified and a photo-identification catalogue to be developed (see below), thus facilitating the confirmation of resightings of dolphins in the net. It was not possible to identify many species of fish from the video footage. The video footage of all 36 trawls was of similar quality and therefore not graded.

The first and last time a dolphin was observed inside and outside the net was recorded to obtain an approximate measure of the time dolphins interacted with the net during a trawl. The camera's field of view was much wider and deeper inside than outside the net (Fig. 3). Estimates of the temporal occurrence of dolphins outside the nets are therefore likely to be underestimated and individual dolphins could not be identified with confidence. Data for dolphins observed outside the net are presented

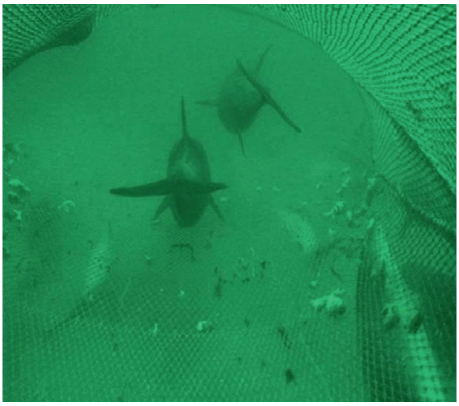

Figure 3. Still image of dolphins inside and outside an actively fishing trawl net, showing field of view of the camera.

here solely for reference and comparison with the more accurate proportions of behaviors recorded inside the net.

Six trawls with dolphins present inside the net (two from each fishing trip) were subsampled using focal individual follows to establish the percentage of the total trawl duration during which individual dolphins were present in the net, their average dive time inside the net and the number of times they returned to the net in each trawl. We also investigated whether the average dive time and number of returns to the net was influenced by the presence of conspecifics inside the net. This was done by analyzing three trawls featuring three different, single individuals entering the net and three trawls during which multiple dolphins (five, eight, and nine individuals) entered the net in groups. In each of the six trawls, a single previously identified individual was observed and followed throughout the duration of the trawl, resulting in six focal follows. The results obtained from this subsample of focal follows were compared with the behavioral events obtained using the scan sampling method described below.

Dolphin Identification and Behavior

Every dolphin that entered the net was identified based on morphological characteristics, such as scars and irregularities of the dorsal fin or fluke, and given a unique identification number. A still image of every dolphin was captured, illustrating the natural markings used to identify the individual and, where possible, its dorsal and ventral aspects. Behavioral data were collected from all focal dolphins present inside and outside the net. If multiple dolphins were present inside the net simultaneously, every dolphin's behavior was analyzed separately and the tape rewound after each focal follow. A number of behavioral events were recorded within three broad behavioral states (traveling, foraging, and socializing) (Table 1). For example, "fish chase" and "fish catch" were two events recorded within the behavioral state "foraging" (Table 1). The following information and attributes were recorded for every behavioral event: date, vessel name, trip number and trawl number, the animal's position in relation to the net, the behavioral event displayed, and comments including

Table 1. Ethogram defining the behavioral states and events recorded in this study of subsurface dolphin behavior in and around trawl nets in the Pilbara Fish Trawl Interim Managed Fishery (PTF).

Behavioral state (bold) and event	Description
**Traveling**	
Entry_head first	Enters the net head first.
Entry_sideways	Enters the net so that left or right side is visible.
Entry_tail first	Enters the net tail first, thus backing down into net.
Exit net	Swims out of the net.
Leave field of view	Dolphin inside the net either swims behind the camera or swims out of view, *e.g.,* if large amounts of sediment are present. Not recorded for dolphins outside the net.
Rest on net	Lies on surface of net for >2 s.
Trampolining	Bounces on external surface of net, one or multiple times, with each bounce <2 s.
**Foraging**	
Head scan	Moves head from side to side.
Fish chase	Chases fish; may or may not result in capture.
Fish catch	Catches fish.
Head shake	Rapidly moves head from side to side with captured fish in mouth.
Inverted foraging	Inverts so that ventrum faces upward while chasing fish.
**Socializing**	
Belly to belly	Two dolphins make belly to belly contact.
Copulation	Dolphins mating or belly to belly for >5 s.
Dolphin bite	Bites another dolphin in social interaction.
Dolphin chase	Chases another dolphin, *e.g.,* out of the net.
Pec fin-pec fin rub	Contact between the pectoral fins of two dolphins.
Social invert	Dolphin inverts presenting its ventrum to another dolphin.

whether or not the animal was resighted. The ID number and the dolphin's sex (if discernible from the ventral aspect) were recorded only for those dolphins that entered the net. Most dolphins that entered the net appeared to be adults; however, the size of individuals could not be measured.

Image Analysis

We used a scan sampling method (Altmann 1974) to quantify the behavioral events exhibited by the dolphins. This method involved detailed sampling for one minute, followed by fast forwarding the imagery for five minutes and repeating this procedure throughout the length of the recording. The results from this scan sampling method were compared with those from analyzing the entire video *via* continuous sampling (Altmann 1974) for two trawls. The proportions of behavioral events recorded were compared between the continuous and scan sampling methods using a Kolmogorov-Smirnov test. This test indicated that the relative frequencies of behavioral events did not differ significantly between the scan and continuous sampling methods (K-S, $D = 0.43$, $P = 0.54$). The more time-efficient scan sampling method was therefore adopted to process all trawls.

Scan sampling was paused and an event recorded when: (1) the first and last dolphin that entered the camera's field of view inside and outside the net did so during

the 5 min fast-forwarding period and (2) the start or end of a trawl fell between the one minute sampling periods. This meant that the estimate of dolphins' temporal association with the nets was not affected by the sampling method.

The duration of a trawl was defined as the time from when the net was fully extended to the time when the net had completely collapsed on reaching the surface (n = 33 trawls), or when the camera stopped recording (n = 3 trawls). This definition allowed the proportion of trawl time that dolphins were present around the net to be calculated, even when recording stopped before the end of a trawl.

Data Analyses

Statistical analyses were performed in PASW Statistics v17. The total number of each behavioral event was recorded and summed for each behavioral state for dolphins inside and outside the net. These data were used to provide a description of the behaviors exhibited by dolphins. The total number of behavioral events, excluding entries and exits into and from the net, was calculated for each dolphin in each trawl. The percentages of events in each behavioral state were calculated for each dolphin in each trawl and then the mean percent of behavioral events and states were calculated separately for each trawl and over all trawls.

Results

Association of Dolphins with Trawl Nets

The mean duration of the 36 analyzed trawls was 2 h 14 min ± 9 min (±1 SE, range of trawl durations = 33 min to 3 h 20 min). Dolphins were observed outside the net in 94% of trawls (n = 34) and entered the net in 81% of trawls (n = 29). Outside the net, they were present for an average of 77% ± 5% of the trawl duration (range = 22%–99%) and inside the net during an average of 59% ± 7% of the total trawl time (range = 2%–98%). A total of 87 entries into the net were recorded, with most dolphins entering head first or sideways (43% for each) and 14% entering tail first, *i.e.,* slowly drifting backward into the net before swimming in the same direction as the trawler and net. No dolphin swam behind the camera in front of the BRD during the 36 trawls analyzed for this study. Three dolphins were observed interacting with the grid on a camera mounted behind the BRD for the purposes of another study (VFJ, SJA, JJM and NRL, unpublished data).

Observations from continuous sampling of six dolphins that entered the net in six separate trawls indicated that dolphins entered the net more often if they were alone in the net (mean ± 1 SE = 11 ± 4 entries, range = 6–19 entries, n = 3 trawls), than if other dolphins were inside the net during that trawl (6 ± 2 entries, range = 3–10 entries, n = 3 trawls). However, the mean presence time of individuals in the net did not differ between group sizes (mean for dolphins alone = 2 min 21 s ± 13 s *cf.* mean for dolphins together = 2 min 15 s ± 15 s). The longest recorded dive time inside the net of any individual was 7 min 2 s; during that dive, no other individuals were present inside the net.

Dolphins were recorded inside and around the net in 34 of the 36 analyzed trawls, regardless of where the vessel operated. A total of 29 individual dolphins were identified from videos recorded inside the net. The number of dolphins

present in the net at the same time ranged from one during most trawls (n = 15 trawls) to seven (n = 1 trawl). The highest cumulative number of individuals observed in the net during a single trawl was nine dolphins. During seven of the 36 trawls, no dolphins were observed inside the net, although dolphins were observed outside the net during five of these trawls. These trawls occurred during different fishing trips and in different fishing areas. During winch-up, the group sizes of dolphins around the stern of the trawler were estimated to range from approximately 25 to 50 dolphins.

The mean number of dolphins in the net per trawl was 2 ± 0.4 (range = 1–9, n = 29). Of the 29 identified individuals, 12 entered the net in only one trawl: 7 of these entered the net only once, while the remaining 5 returned to the net multiple times during the trawl. A further 10 dolphins were each resighted in either two or three different trawls during the same fishing trip. Seven dolphins were sighted repeatedly inside the net in different fishing trips (Fig. 1). One of these individuals entered the net during all three trips (Fig. 1). This suspected male was also the individual with the highest number of resightings; it was seen during a total of nine trawls. The remaining six individuals were each sighted in two of the three trips and in all of the areas where trawling occurs (Fig. 1). Three of these individuals were observed in one area only—areas 2, 4, and 5, respectively, while the other four dolphins entered the net in two areas each (Fig. 1). Three of these four dolphins were recorded when the vessel was fishing close to the border between two areas. Nine dolphins were repeatedly observed in groups of two, either in different trawls of the same trip or during different trips.

Dolphin Behavior

A total of 1,142 behavioral events were recorded from the scan sampling of 36 trawls, with 406 events recorded from dolphins inside the net and 736 events from those outside the net. Inside the net, dolphins displayed a wider variety of behaviors overall (14 types of events) in each behavioral state (traveling [5], foraging [5], socializing [4]) than dolphins outside the net. The total number of events recorded, excluding entries into (86) and exits from (63) the net, was 993.

A total of 257 events were recorded from dolphins inside the net, with 221 of these classified as foraging (86%) and 36 as socializing (14%). When entries and exits were excluded, behavioral events were recorded from 24 trawls inside the net. The highest mean proportions of behaviors inside the net were foraging behaviors (88% ± 4.8%), followed by socializing (12% ± 4.8%). The main foraging behaviors were chasing fish, scanning for fish and catching fish, while chasing other dolphins was the most common socializing event inside the net (Fig. 4).

Outside the net, where the field of view was limited and individuals could not be identified, dolphins were present during 29 trawls. The most common behavioral state was traveling (mean = 63.3% ± 5.1% of events per dolphin per trawl), followed by foraging (mean = 34.6% ± 5.2%) (Fig. 4). Trampolining, classified in this study as a traveling behavior, dominated the traveling events recorded outside the net (*ca.* 60%, Fig. 4).

Social behaviors were relatively rare, with a total of 36 events recorded inside the net and only five events recorded outside the net (Fig. 4). The most common social event recorded both inside and outside the net was chasing dolphins, followed by

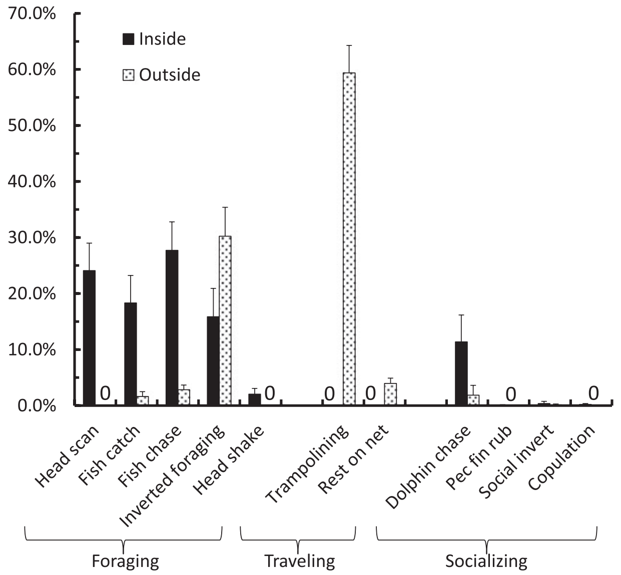

Figure 4. Mean percentages of behavioral events within behavioral states per dolphin per trawl (±1 SE), recorded from video observations of dolphins inside and outside actively fishing trawl nets in the Pilbara Trawl Fishery. 0 = no event recorded.

social inverting, where an individual inside the net inverted to present its ventrum to a dolphin outside the net, or two dolphins outside the net presented their ventra to each other, whereby one dolphin inverted.

Discussion

Association of Dolphins with Fishing Gear

This study of the subsurface behavior of bottlenose dolphins around operating fish trawl nets is one of the few visual underwater assessments of dolphin-trawler associations. Previous studies have assessed delphinid subsurface behavior around trawl gear, but in less detail and without being able to identify individuals (Broadhurst 1998, Mackay 2011). We documented high interaction rates between dolphins and trawl nets in the Pilbara Trawl Fishery (PTF). Dolphins were present outside the net in 94% of all assessed trawls and for up to 99% of the duration of an individual trawl, while they entered the net during 81% of all trawls and were present inside the net for up to 98% of the trawl duration. These interaction rates are higher than those previously reported by Mackay (2011), who recorded the presence of dolphins inside the net during 66% of all trawls and for up to 64% of the trawl duration. However, she noted that the interaction rates in her study were likely to be underestimated, as it was not possible to assess entire trawls.

It is difficult to draw comparisons between this study and other assessments of dolphin-trawler interactions, since they have focused primarily on dolphin behavior at the surface or the damage to target catch and gear caused by dolphins (*e.g.,* Chilvers and Corkeron 2001, Gonzalvo *et al.* 2008). Interaction rates between dolphins and

trawl fisheries may be higher than can be determined from observations made from the surface or upon retrieving the catch. Interactions which occur while the trawl net is actively fishing may lead to unobserved bycatch if dolphins asphyxiate in the net during trawling and are expelled through the BRD's escape hatch before the net is retrieved. This is likely to have important conservation implications for dolphin communities that frequently interact with trawl fisheries, as the rate of injury and mortality in trawl nets is likely to be higher than that which is observed from onboard the trawl vessels.

Furthermore, most reports of the interactions between dolphins and trawlers have focused on opportunistic feeding by dolphins on enmeshed fish during winch-up or discarded bycatch around trawlers (Corkeron *et al.* 1990, Fertl and Leatherwood 1997, Dahlheim and Heyning 1999, Bearzi 2002), while few studies have quantified dolphin behavior in and around actively trawling nets. Our study demonstrates that bottlenose dolphins associating with trawl vessels in the Pilbara actively forage within and around the nets while they are fishing, and also take advantage of discards after winch-up.

Subsurface Behavior of Dolphins

Dolphins displayed a high proportion of foraging behaviors both inside and outside the actively fishing trawl net. Dolphins that enter the net are likely to do so for the opportunities of encountering large numbers of potential prey, but also because the net's surface provides a barrier against which dolphins can chase and catch fish. Fish chased by dolphins often swam into the meshes, where they became entangled and were easily captured by the dolphins.

Dolphins also foraged on the outside of the net. The main foraging behavior recorded was inverting to swim underneath the net (n = 186). This suggests that some fish swim underneath the net when it is not in contact with the sea floor, providing dolphins outside the net with a food source that may not be found in similar proportions near the outer sides or upper surface of the net. Occasionally, dolphins were observed pulling enmeshed fish from the net, a behavior previously observed around codends in Australian prawn trawl fisheries (Broadhurst 1998, Svane 2005). While our cameras did not capture footage of the area around the codend, dolphins in the PTF are also likely to take prey from that section of the net. The current study indicated that trawl vessels operating in the PTF present bottlenose dolphins with numerous foraging opportunities beyond that of feeding on discards after the catch is sorted.

The most common behavior observed outside the net was trampolining, which we classified as a traveling behavior, since dolphins that exhibited the behavior frequently moved forward towards the net opening while performing a series of bounces on the net. This behavior, to the authors' knowledge, has not been previously recorded in any other fishery and did not appear to contribute to socializing, nor to any other commonly used behavioral category. Trampolining dolphins often turned and twisted their bodies when bouncing on the net as it moved through the water, and trampolining was sometimes preceded, or followed, by head and rostrum rubbing against the net. Trampolining may therefore be performed to remove old skin, parasites, or even remoras that were observed on three individuals. Since many delphinids have a tendency to investigate and interact with physical and biological features in their environment (*e.g.,* Jacobsen 1986, Shane *et al.* 1986, Mann and Smuts 1999), trampolining may also simply be a play behavior.

Our results indicate that dolphins exploit trawl nets for more than just foraging opportunities. The motivating factors behind dolphin interactions with trawl nets highlight the importance of further video camera deployments to determine which areas of the nets present the greatest risk of entanglement to dolphins. Although the rate of dolphin bycatch is relatively low in the PTF (<1 dolphin per 100 trawls, range = 0.36–0.91, depending on whether skipper logbook or observer data are used to estimate the annual bycatch), this extrapolates to an incidental capture of about 17–50 dolphins per year (Allen and Loneragan 2010).

The downward-opening hatch currently used in the PTF is unlikely to allow dolphins to escape to the surface. All dolphins observed inside the trawl net, and almost all those observed in the previous study by Mackay (2011), were swimming in the same direction as the vessel (Fig. 3). This observation, and the responses of three dolphins captured on a camera mounted behind the BRD (VFJ, SJA, JJM and NRL, unpublished data), suggest that dolphins are likely to swim forward and upward when coming into contact with the bycatch exclusion grid and may not be able to locate the escape hatch at the bottom of the net.

Acoustic harassment devices or pingers, used to alert animals to the presence of fishing gear, do not appear to be effective deterrents for bottlenose dolphins (*Tursiops* spp.) interacting with static fishing nets, *e.g.,* gill nets, due to this species' tendency to habituate to the associated "dinner bell effect" (Dawson *et al.* 1998, Cox *et al.* 2003, Brotons *et al.* 2008). Pingers were also proven to be ineffective in the PTF (Stephenson and Wells 2006). Recent trials of larger, louder devices appeared to reduce the bycatch of harbor porpoises (*Phocoena phocoena*) in static nets and that of common dolphins (*Delpinus delphis*) in a pelagic pair trawl fishery in the North Sea (Northridge *et al.* 2011). The authors cautioned, however, that sample sizes for the trials involving dolphins remain too small to provide statistically robust evidence of their efficacy (Northridge *et al.* 2011). Modifications to fishing nets and BRDs may offer the most effective solution for reducing delphinid bycatch, as they are less costly than effort reductions or spatial/temporal closures, and have been successful in reducing dolphin capture and mortality in other circumstances (*e.g.,* Hall *et al.* 2000).

Specialization Within a Community of Trawler-associated Dolphins?

About 25–50 dolphins were observed around the trawlers when the nets were hauled. These numbers are likely to represent a relatively small proportion of the total population of dolphins that inhabit the area trawled by the PTF (12,779 km^2). The relatively small numbers of dolphins observed around trawl vessels suggests that they may form a community within a larger population of unknown size that inhabits the region. A study from Moreton Bay, Queensland, identified two dolphin communities within a broader population: one that fed in association with trawlers and another that did not (Chilvers and Corkeron 2001). The two communities differed in group sizes and habitat preference and were socially segregated (Chilvers and Corkeron 2001). Whether a similar scenario occurs in the PTF requires testing using photo-identification and genetic markers, which forms the basis of current studies (SJA, unpublished data).

Furthermore, the number of individual dolphins recorded inside the nets (just 29 identified individuals in total and a maximum of nine in any one trawl) relative to the 25–50 dolphins observed at the surface around the vessels after each trawl, suggests that entering the nets to forage may represent a specialized behavior. This specialization may be exhibited by a limited number of individuals within the

community of trawler-associated dolphins, a hypothesis supported by the observation that dolphins were observed outside the net during five trawls, but none of them entered the net. Similarly, the fact that 22 of the 29 dolphins that entered the net did so a number of times during the same trawl, suggests that these individuals spent little or no time interacting with the outside of the net, but left it only to breathe at the surface before returning into the net. With the exception of one individual, all of the seven dolphins that were resighted during different trawls entered the trawl nets within the same localized area (Fig. 1). This suggests that, while dolphins have the ability to follow vessels throughout the fishing grounds, they appear to interact with trawl nets opportunistically when a trawler is present within a certain area. Foraging traditions that are restricted to particular groups or matrilines have been documented in several other bottlenose dolphin populations (Chilvers and Corkeron 2001, Mann and Sargeant 2003).

Implications for Reducing the Fishing-related Mortality of Dolphins

Underwater video analyses of the interactions between dolphins and exclusion grids during capture events are critical for evaluating the effectiveness of the bycatch reduction devices used in the PTF. Industry and the Western Australian Department of Fisheries have recently reinitiated deployments of video cameras inside and outside trawl nets in the PTF to record the bycatch events known to occur from observer accounts. The threat posed to the resident dolphin population by past and ongoing dolphin bycatch in this fishery, however, cannot be fully quantified until genetic data and population abundance estimates become available. In view of the high interaction rates recorded inside trawl nets in this study, mitigation efforts to reduce dolphin bycatch and mortality should focus on preventing dolphins from becoming caught, injured or killed in the gear, rather than attempting to prevent these individuals from interacting with the nets. This may be achieved through the development of more effective bycatch reduction devices in this fishery.

Acknowledgments

This research was funded by a Fisheries Research and Development Corporation grant (FRDC 2008/048), the Western Australian Department of Fisheries, the Nickol Bay Professional Fishers Association, and the Thyne Reid ECOCEAN scholarship to VFJ. We thank the FRDC project Steering Committee of Lindsay Joll, Graeme Stewart, and Shane O'Donoghue for their advice and guidance during the project. The research was carried out under wildlife research permits from the Western Australian Department of Environment and Conservation and the Commonwealth Department of Environment, Water, Heritage and the Arts, and animal ethics approval from Murdoch University (W2182/08). Sincere thanks to Gavin Kewan for collection of video footage and observer data on board trawl vessels, and to the skipper and crew of the vessel from which we worked for their hospitality. We thank Corey Wakefield and Julian Tyne for helping to produce Figures 1 and 2, respectively, and Giovanni Bearzi, Brett Molony, and two anonymous reviewers for comments that improved this manuscript.

Literature Cited

Allen, S., and N. Loneragan. 2010. Reducing dolphin bycatch in the Pilbara Finfish Trawl Fishery. Final Report to the Fisheries Research and Development Corporation (FRDC). Murdoch University, Perth, Australia. 59 pp. ISBN: 978-0-86905-926-5.

Altmann, J. 1974. Observational study of behavior: Sampling methods. Behaviour 49:227–267.

Bearzi, G. 2002. Interactions between cetaceans and fisheries in the Mediterranean Sea. *In* G. Notarbartolo Di Sciara, ed. Cetaceans of the Mediterranean and Black Seas: State of knowledge and conservation strategies. A report to the ACCOBAMS Secretariat, Monaco. Section 9. 20 pp. Available at http://theblackfish.org/files/driftnets_cetaceans_report.pdf.

Bearzi, G., C. M. Fortuna and R. R. Reeves. 2008. Ecology and conservation of common bottlenose dolphins *Tursiops truncatus* in the Mediterranean Sea. Mammal Review 39: 92–123.

Broadhurst, M. K. 1998. Bottlenose dolphins, *Tursiops truncatus*, removing bycatch from prawn trawl codends during fishing in New South Wales, Australia. Marine Fisheries Review 60:9–14.

Brotons, J., Z. Munilla, A. Grau and L. Rendell. 2008. Do pingers reduce interactions between bottlenose dolphins and nets around the Balearic Islands? Endangered Species Research 5:301–308.

Chilvers, B. L., and P. J. Corkeron. 2001. Trawling and bottlenose dolphins' social structure. Proceedings of the Royal Society London 268:1901–1905.

Corkeron, P. J., M. M. Bryden and K. E. Hedstrom. 1990. Feeding by bottlenose dolphins in association with trawling operations in Moreton Bay, Australia. Pages 329–336 *in* S. Leatherwood and R. R. Reeves, eds. The bottlenose dolphin. Academic Press, San Diego, CA.

Couperus, A. S. 1997. Interactions between Dutch midwater-trawl and Atlantic white-sided dolphins (*Lagenorhynchus acutus*) southwest of Ireland. Journal of Northwest Atlantic Fishery Science 22:209–218.

Cox, T. M., A. J. Read, D. Swanner, K. Urian and D. Waples. 2003. Behavioral responses of bottlenose dolphins, *Tursiops truncatus*, to gillnets and acoustic alarms. Biological Conservation 115:203–212.

Dahlheim, M. E., and J. E. Heyning. 1999. Killer whale—*Orcinus orca* (Linnaeus, 1758). Pages 281–322 *in* S. H. Ridgway and S. R. Harrison, eds. Handbook of marine mammals. Volume 6. The second book of dolphins and porpoises. Academic Press, London, U.K.

Dalla Rosa, L., and E. R. Secchi. 2007. Killer whale (*Orcinus orca*) interactions with the tuna and swordfish longline fishery off southern and south-eastern Brazil: A comparison with shark interactions. Journal of the Marine Biological Association of the United Kingdom 87:135–140.

Dawson, S. M., A. Read and E. Slooten. 1998. Pingers, porpoises and power: Uncertainties with using pingers to reduce bycatch of small cetaceans. Biological Conservation 84:141–146.

Dawson, S., F. Pichler, E. Slooten, K. Russell and C. S. Baker. 2001. The North Island Hector's dolphin is vulnerable to extinction. Marine Mammal Science 17:366–371.

Department of Fisheries. 2011. State of the Fisheries and Aquatic Resources Report 2010/11. W. J. Fletcher and K. Santoro, eds. Department of Fisheries, Western Australia. 359 pp. Available at http://www.fish.wa.gov.au/About-Us/Publications/Pages/Annual-Report.aspx.

Diaz-Lopez, B., L. Marini and F. Polo. 2005. The impact of a fish farm on a bottlenose dolphin population in the Mediterranean Sea. Thalassas 21:65–70.

Fertl, D., and S. Leatherwood. 1997. Cetacean interactions with trawls: A preliminary review. Journal of Northwest Atlantic Fishery Science 22:219–248.

Gonzalvo, J., M. Valls, L. Cardona and A. Aguilar. 2008. Factors determining the interaction between common bottlenose dolphins and bottom trawlers off the Balearic Archipelago (western Mediterranean Sea). Journal of Experimental Marine Biology and Ecology 367:47–52.

Hall, M. A. 1998. An ecological view of the tuna-dolphin problem: Impacts and trade-offs. Reviews in Fish Biology and Fisheries 8:1–34.

Hall, M. A., D. L. Alverson and K. I. Metuzals. 2000. By-catch: Problems and solutions. Marine Pollution Bulletin 41:204–219.

Hill, B. J., and T. J. Wassenberg. 1990. Fate of discards from prawn trawlers in Torres Strait. Australian Journal of Marine and Freshwater Research 41:53–64.

Jacobsen, J. K. 1986. The behaviour of *Orcinus orca* in Johnstone Strait, British Columbia. Pages 135–185 *in* B. C. Kirkevold and J. S. Lockard, eds. Behavioral biology of killer whales. John Wiley & Sons, New York, NY.

Mackay, A. 2011. An investigation of factors related to the bycatch of small cetaceans in fishing gear. Ph.D. thesis, University of St Andrews, St Andrews, U.K. 317 pp. Available at http://research-repository.st-andrews.ac.uk/handle/10023/1888.

Mann, J., and B. Sargeant. 2003. Like mother like calf: The ontogeny of foraging traditions in wild Indian Ocean bottlenose dolphins (*Tursiops* sp.). Pages 236–266 *in* D. M. Fragaszi and S. Perry, eds. The biology of traditions: Models and evidence. Cambridge University Press, Cambridge, U.K.

Mann, J., and B. Smuts. 1999. Behavioral development in wild bottlenose dolphin newborns (*Tursiops* sp.). Behaviour 136:529–566.

Newman, S. J., G. A. Hyndes, J. W. Penn, M. C. Mackie and P. C. Stephenson. 2003. Review of generic no-take areas and conventional fishery closure systems and their application to the management of tropical fishery resources along North-Western Australia. Pages 75–85 *in* Proceedings of the World Congress on Aquatic Protected Areas, Cairns, Australia, August 2002.

Nitta, E., and J. Henderson. 1993. A review of interactions between Hawaii's fisheries and protected species. Marine Fisheries Review 55:83–92.

Northridge, S., A. Kingston, A. Mackay and M. Lonergan. 2011. Bycatch of vulnerable species: Understanding the process and mitigating the impacts. Final Report to Defra Marine and Fisheries Science Unit, Project no MF1003. University of St Andrews. Defra, London, U.K. 99 pp. Available at http://sciencesearch.defra.gov.uk/Document.aspx?Document=MF1003-FINALRevisedAugust2011.pdf.

Read, A. J. 2008. The looming crisis: Interactions between marine mammals and fisheries. Journal of Mammalogy 89:541–548.

Read, A. J., D. M. Waples, K. W. Urian and D. Swanner. 2003. Fine-scale behaviour of bottlenose dolphins around gillnets. Proceedings: Biological Sciences 270:S90–S92.

Rogan, E., and M. Mackey. 2007. Megafauna bycatch in drift nets for albacore tuna (*Thunnus alalunga*) in the NE Atlantic. Fisheries Research 86:6–14.

Rojas-Bracho, L., R. R. Reeves and A. Jaramillo-Legorreta. 2006. Conservation of the vaquita *Phocoena sinus*. Mammal Review 36:179–216.

Seager, J. 2008. EventMeasure Version 2.04. SeaGIS Pty. Ltd., Bacchus Marsh, Victoria, Australia. Available at http://www.seagis.com.au.

Shane, S., R. Wells and B. Würsig. 1986. Ecology, behavior and social organization of the bottlenose dolphin: A review. Marine Mammal Science 2:34–63.

Stephenson, P. C., and S. Wells. 2006. Evaluation of the effectiveness of reducing dolphin catches with pingers and exclusion grids in the Pilbara trawl fishery. Final report to Fisheries Research and Development Corporation on Project No. 2004/068 Fisheries Research Report No 173, Department of Fisheries, Western Australia. 44 pp.

Stephenson, P. C., S. Wells and J. A. King. 2006. Evaluation of exclusion grids to reduce the catch of dolphins, turtles, sharks and rays in the Pilbara trawl fishery. DBIF Funded Project. Fisheries Research Report No. 171, Department of Fisheries, Western Australia. 24 pp.

Svane, I. 2005. Occurrence of dolphins and seabirds and their consumption of by-catch during prawn trawling in Spencer Gulf, South Australia. Fisheries Research 76:317–327.

Waring, G. T., P. Gerrior, P. M. Payne, B. L. Parry and J. R. Nicolas. 1990. Incidental take of marine mammals in foreign fishery activities off the northeast United States, 1977–88. Fishery Bulletin 88:347–360.

Zeeberg, J. J., A. Corten and E. De Graaf. 2006. Bycatch and release of pelagic megafauna in industrial trawler fisheries off Northwest Africa. Fisheries Research 78:186–195.

Received: 27 June 2011
Accepted: 14 August 2012
